# Feasibility of a comprehensive medication review to improve medication use for patients with cancer and comorbid conditions

**DOI:** 10.1007/s00520-022-07413-8

**Published:** 2022-10-20

**Authors:** Emily R. Mackler, Michelle K. Azar, Emily Johengen, Karen B. Farris, Amy N. Thompson

**Affiliations:** 1 Michigan Oncology Quality Consortium (MOQC) and Michigan Institute for Care Management and Transformation (MICMT), 4251 Plymouth Road Arbor Lakes, Building 3, Floor 3, Ann Arbor, MI 48105 USA; 2 Johns Hopkins Department of Pharmacy, Baltimore, MD USA; 3 Trinity Health IHA Medical Group Hematology Oncology, Ann Arbor, MI USA; 4 College of Pharmacy, University of Michigan, Ann Arbor, MI USA

**Keywords:** Comorbidities in cancer, Polypharmacy, Interdisciplinary, Survivorship

## Abstract

**Purpose:**

A focus on oral medications for patients receiving care from both oncologists and primary care providers elicits an opportunity for improvement in patient outcomes. The purpose of this pilot study was to explore the feasibility and appropriateness of a comprehensive medication review (CMR) by a primary care pharmacist in a population of patients with cancer and chronic conditions.

**Methods:**

Adult patients who received both cancer and primary care at Michigan Medicine, received active systemic cancer treatment, and had a comorbid condition of diabetes, hypertension, chronic heart failure, depression, and/or anxiety were eligible to receive a CMR by the primary care clinical pharmacist. Data collected included number eligible for the CMR (feasibility), patient demographics, medication-related problems (MRPs) and medication interventions (appropriate), number of patients requiring follow-up with the clinical pharmacist or physician, and pre/post-intervention changes in A1c and BP, as applicable.

**Results:**

Of the 96 patients that met inclusion criteria, 55 patients (57%) received a CMR. Pharmacists provided 66 instances of patient education and identified 22 medication-related problems (MRPs) in 15 (27%) of patients. After CMRs were completed, 22 patients (40%) were referred to primary care pharmacists or physician providers for ongoing care.

**Conclusion:**

A CMR was feasible and appropriate for patients with chronic conditions receiving treatment for cancer.

## Introduction

Concomitant chronic medical conditions can affect the care of patients with cancer. Among Medicare beneficiaries with cancer, 40% have at least one comorbid condition and 15% have two or more [1]. The most common of these include cardiovascular disease, diabetes mellitus, and mental health disorders [1]. Several studies have shown that patients with comorbid conditions are less likely to be offered chemotherapy or surgery, less likely to complete a treatment regimen, and more likely to receive chemotherapy at a reduced dose if it is offered [2]. In some cases, the decision to deviate from standard treatment is the result of a risk-based discussion between the patient and care team with the goal of limiting toxicity.

Many oral anticancer agents (OAAs) that are taken for years present challenges such as drug-drug interactions (DDIs) and side effects that worsen chronic conditions [1, 3]. For example, the vascular endothelial growth factor (VEGF) inhibitors are a class of intravenous and oral medications that are used to treat late-stage solid tumors. VEGF inhibitors can cause new or worsening hypertension, as well as nephropathy, specifically proteinuria, and can worsen renal function in patients with chronic kidney disease [4]. Studies estimate that potential DDIs affect about one-third of patients treated for cancer, and hospitalizations for adverse drug reactions (ADRs) are more common when patients are prescribed multiple, potentially interacting medications [5–7]. Rates of DDIs and ADRs have likely increased since these studies were published due to the increased use of long-term OAAs and the rates of comorbid conditions in the aging cancer population.

The rising importance of comorbid conditions in patients with cancer has elucidated a need for better communication between primary care providers (PCPs) and oncology specialists. Multiple appeals for improved coordination of care have been published, highlighting a need for more specific designation of roles, increased communication of treatment plan changes, and discussions regarding goals of care [8–14].

The purpose of this pilot study was to test whether pharmacist-led comprehensive medication reviews (CMRs) for cancer patients with comorbid conditions were feasible and appropriate. This pilot study was conducted through collaboration of Michigan Medicine with the Michigan Oncology Quality Consortium (MOQC) [15], a continuous quality improvement collaborative which seeks to improve oncology care in Michigan.

## Methods

### Design/participants

We conducted a single-center, observational, quality improvement pilot study. Patients ≥ 18 years old receiving active systemic cancer treatment from 10/31/2016 to 10/31/2017, and those with established primary care at one of Michigan Medicine’s general medicine clinics, defined as having 1 patient visit between January 2017 and June 2018, were eligible. Patients were screened and included if they had one or more comorbidities, including hypertension, diabetes, heart failure, depression, and/or anxiety.

### Intervention

The pharmacist performing the CMR was an embedded primary care pharmacist with a collaborative practice agreement for chronic disease management at Michigan Medicine, a MOQC site [16]. Eligible patients received a CMR in clinic or via telephone from the primary care pharmacist. Following CMRs, pharmacists worked with physicians and patients to manage clinical care via patient education and motivational interviewing [17], medication adjustments, and care coordination.

### Data collection

Data collected from the CMR included patient demographics, medication-related problems (MRPs), which were categorized by adherence, safety, indication, and effectiveness [18, 19], medication interventions including number of medications added, deleted, or changed to the medication list, number of patients referred for physician or pharmacist follow-up, and pre/post-intervention changes in A1c and BP (as applicable). All patients received one visit with the pharmacist with subsequent follow-up determined by CMR findings. This quality improvement project was deemed nonregulated by the University of Michigan Institutional Review Board.

### Analysis

The numbers of eligible participants were determined (feasibility) and MRPs and interventions were described (appropriate).

## Results

### Sample characteristics

Five thousand nine hundred forty-one patients received active cancer treatment, 96 received primary care at our pilot site in Michigan Medicine and had at least one chronic comorbid condition. Of the 96 patients that met inclusion criteria, 55 (57%) received a CMR. Patients were primarily white (70.8%), female (61.5%), and an average of 65 years (Table 1). The primary reason individuals did not receive a CMR was that they could not be reached via telephone with three attempts (32%) (Table 1).

**Table 1:** Patient demographics

	Number (%)	
Patients contacted	96	
Completed	55 (57)	
Unable to reach after 3 attempts	30 (32)	
Refused	6 (6)	
Deceased	4 (4)	
Primary care established outside of pilot clinic	1 (1)	
Demographic information	Total cohort = 96 Number (%)	Completed CMRs = 55 Number (%)
Mean age in years (± SD)	65 (± 12.3)	66 (± 11.5)
Female gender	59 (61.5)	32 (58.2)
Race		
Black White	18 (18.8) 68 (70.8)	15 (27.3) 37 (67.3)
Mean # of meds (± SD)	10 (± 6.3)	11 (± 5.4)
Mean # of concomitant chronic conditions, including pain	2.2 (± 0.95)	2.4 (± 0.94)
Hypertension	69 (71.9)	40 (72.7)
Diabetes	22 (22.3)	14 (25.5)
Congestive heart failure	11 (11.5)	7 (12.7)
Depression or anxiety	40 (42.7)	23 (41.8)
Previous pain diagnosis	66 (68.8)	42 (76.4)
On an anticoagulant	39 (40.6)	27 (49)

### Pharmacist interventions

After CMRs, 44 (80%) patients had changes to their medication list and 22 (40%) required additional follow-up. Pharmacists provided 66 instances of patient education for medications, lifestyle, and disease monitoring. Additionally, 22 MRPs were identified in 15 (27%) patients (Table 2). Adverse events, unaddressed indication, patient adherence, and affordability were identified as problems. Notably, the medication burden among those receiving a CMR was high, with an average of 10 medications/patient. For patients requiring additional follow-up, 10 (18%) were referred to primary care pharmacists for chronic disease management and 12 (22%) required follow-up with a physician provider, 10 to PCP and 2 to specialty (cardiology and oncology). Of note, 2 patients referred to their PCP had not been seen in over a year (14 and 22 months, respectively). Other reasons for referral to physicians included need for follow-up assessment of acute issues such as vision changes and pain. A patient case example can be found in Table 3. There were no significant changes in clinical outcomes measured (Table 2). Based upon the MRPs, a CMR is an appropriate intervention for patients receiving active cancer treatment and with comorbid conditions.

**Table 2:** Results from CMR

Interventions by pharmacist	*N* (%)
Referred to for additional follow-up	22 (40)
Physician	12 (22)
PharmD	10 (18)
Medication-related problems identified	22
Adherence	7
Non-adherence	3
More cost-effective medication available	1
Patient unable to afford medication	3
Safety	5
Medication interaction	1
Undesirable (adverse) effect	3
Incorrect administration	1
Unaddressed Indication	3
Needs additional therapy	3
Effectiveness	2
More effective medication available	1
Dosage form inappropriate	1
Other	5
Medication list updated	44 (80)
Instances of patient education provided	66
Average reduction in systolic BP*	4 mm Hg (± 19.6)
Average reduction in diastolic BP*	3 mm Hg (± 10.9)
Average A1c at point 1 vs point 2**	6.7 (± 0.9) vs 6.9 (± 1.4)

^*^Patients with diagnosis of hypertension. **Patients with diagnosis of diabetes

**Table 3:** Patient case

The patient is a 77-year-old white female with a history of metastatic endometrioid carcinoma, receiving treatment with carboplatin and docetaxel	
Past medical history: • Deep vein thrombosis – diagnosed 7 months prior • Hypertension • Type 2 diabetes • Anxiety • Ruptured disk • Non-alcoholic steatohepatitis	Current medications: • Glipizide 5 mg daily • Enoxaparin 60 mg SC BID • Cetirizine 5 mg daily • Gabapentin 300 mg TID • Losartan 25 mg daily • Verapamil SR 240 mg daily • Omeprazole 20 mg daily • Oxycontin CR 10 mg BID • Oxycodone 10 mg q 4 h • Cyclobenzaprine 10 mg TID prn • Docusate sodium 100 mg BID prn • Chemotherapy as mentioned above
Recent hemoglobin A1c was well controlled at 6.1% BP well controlled with a recent BP of 119/54	
Through the CMR process, the patient endorsed significant frustration with enoxaparin and reported her PCP felt it was necessary due to her cancer. Additionally, she reported home blood sugars ranging from 60 to 110 fasting with positive symptoms of hypoglycemia and expressed an interest in physical therapy within her home as she was no longer able to make it to her appointments. Upon visit completion, the pharmacist facilitated discussion with the PCP and oncologist to enable transition from enoxaparin to a direct oral anticoagulant. The pharmacist was able to discontinue glipizide therapy and follow-up with the patient to ensure there were no further hypoglycemic episodes. Finally, through conversations with the PCP, the pharmacist facilitated an order for home physical therapy	

## Discussion

In this quality improvement pilot study, the implementation of a pharmacist-led CMR intervention for patients with cancer and chronic comorbid conditions was feasible and appropriate, as most individuals had multiple recommendations for therapy optimization and enhanced coordination of care. Pharmacists are skilled at identifying DDIs, recognizing medication adverse effects, ensuring appropriateness of therapy, suggesting more cost-effective or patient convenient medications, and improving medication adherence [20]. This pilot identified several areas where disease management and supportive care could be improved, and two of five in this small sample required additional management following the CMR, illustrating the need for medication-focused care coordination with this population.

A recent review of patient perspectives on primary care and oncology coordination identified four key areas that were necessary for patient-centered care coordination. These constructs included communication, defining provider care roles, information access, and individualized patient care [21]. This pilot addresses all four areas and is a model for identifying and improving the care of medically complicated patients with active cancer treatment—with particular attention to those with coexisting chronic conditions—in the future. Pharmacists may be one important option to enhance the coordination of care between primary care and oncology.

Given the aforementioned increased use of long-term anticancer treatment and the rates of comorbid conditions in the aging cancer population, the age of our study population (mean = 65 years) and number of medications (mean = 11) is of great importance. Several publications have highlighted the medication-related risks and resultant toxicity in older patients with cancer who receive multiple medications [22–24]. Notably, a recent study by Dr. Lu-Yao and colleagues demonstrated the relationship between polypharmacy (≥ 5 concurrent medications) and rates of inpatient hospitalization among older adults (> 65 years) treated with chemotherapy. Furthermore, patients with at least 10 prescriptions had a 50% higher rate of post-chemotherapy inpatient hospitalization compared to those with fewer than five medications [23]. Given the consequences of polypharmacy in older patients with cancer, an international guide for conducting medication reviews in this high-risk population was recently developed by a Young International Society of Geriatric Oncology and Nursing and Allied Health Interest Group. This guide recommends adult patients who are starting chemotherapy and on ≥ 5 medications receive a medication review [25].

Despite the potential benefits, several limitations exist. First, as a single-institution study, these results may not be generalizable to other healthcare systems or practices. Of importance, patients in this pilot received both their cancer and primary care within the same system and a shared medical record. We expect that patients who receive their care from different systems would have even greater need for this intervention given the presumed decrease in communication fluidity between providers. In addition, this model of care may not be generalizable to smaller practices where access to primary care pharmacists, or pharmacists in general, may be limited. The completion rate of this intervention was just under 60% with approximately 30% of patients unreachable by the 3^rd^ attempt. Several reasons could exist to account for this high number including patients not being educated in advance that they would receive a phone call offering a CMR, not having a pre-existing relationship with the pharmacist in the primary care clinic, and potentially experiencing fatigue from multiple healthcare encounters. Finally, although we noted improvement in BP control, both larger sample size and longer follow-up are necessary to discern whether there are significant improvements in clinical outcomes.

## Conclusion

A CMR was feasible and appropriate for patients receiving treatment for cancer who also have chronic comorbid conditions and follows recommendations for medication reviews in older adults with cancer and polypharmacy.

## Data Availability

The authors confirm that the data supporting the findings of this study are available within the article.

## Abbreviations

*ADR*: Adverse drug reaction
*BID*: Twice daily
*BP*: Blood pressure
*CMR*: Comprehensive medication review
*DDI*: Drug-drug interactions
*MCC*: Multiple chronic conditions
*MOQC*: Michigan Oncology Quality Consortium
*MRP*: Medication-related problem
*OAA*: Oral anticancer agent
*PCP*: Primary care provider
*SC*: Subcutaneous
*TID*: Three times daily
*VEGF*: Vascular endothelial growth factor
